# The complete chloroplast genome of *Adenophora triphylla* (Asterales: *Campanulaceae*)

**DOI:** 10.1080/23802359.2020.1847613

**Published:** 2021-01-13

**Authors:** Seon-Hwa Bae, Chang-Kug Kim

**Affiliations:** a Department of Horticulture, Institute of Agricultural Sciences and Technology, Jeonbuk National University, Jeonju, Korea; b Genomics Division, National Institute of Agricultural Sciences, Jeonju, Korea

**Keywords:** *Adenophora triphylla*, *Campanulaceae* family, chloroplast genome

## Abstract

*Adenophora triphylla* (*A. triphylla*) is an important oriental herb belonging to the *Campanulaceae* family. *A. triphylla* complete chloroplast genome is composed of 239,431 bp, which form a large single-copy region (LSC, 178,906 bp), a small single-copy region (SSC, 55,819 bp), and 2 inverted repeats (IRs, 2,353 bp). There are 108 genes annotated, including 74 protein-coding genes, 4 ribosomal RNA genes, and 30 transfer RNA genes. Phylogeny indicates that *A. triphylla* was belong to *Adenophora* genus as a sister group and most closely related to *Adenophora divaricate.*

## Main

*Adenophora triphylla* is a perennial herb, which is mainly distributed in China, Japan, Korea, and Russia. It is a flowering plant in the family *Campanulaceae* that is one of the 62 species of *Adenophora* (Kim et al. 2009). *A. triphylla* has been an important medicinal plant in oriental medicine for protect against human obesity, cancer, and inflammation (Chun et al. 2014). *A. triphylla* was collected from National Institute of Horticultural and Herbal Science (NIHHS, geographic coordinate: N36°94′38″, E127°75′33″) and deposited under the NIHHS as voucher number JA2019017.

Genomic DNA was isolated from young leaves using a modified CTAB method (Allen et al. 2006) or Kit for Illumina sequencing and Calson buffer + QIAGEN genomic-tip 500/G Kit. for Oxford Nanopore Technologies (ONT) sequencing. and was subjected to constructing an Illumina paired-end (PE) with 300-bp insert size and ONT libraries unperformed size selection, by following the manufacturer’s instruction. The Illumina PE library was sequenced using an Illumina sequencing platform (Illumina, CA, USA), and ONT library was sequenced using a Nanopore GridION platform (Oxford Nanopore Technologies, Oxford Park, UK). A total of 123.6 Gbp ONT data was mapped to *Adenophora triphylla* chloroplast genome (NCBI accession number, MG764158) as a reference sequence. The mapped 19.4 Gbp reads were performed self-correction, and *de novo* assembly was conducted using a Canu 2.0 program (Koren et al. 2017). The assembled contigs were polished using medaka 1.0 and pilon 1.22 tool (Walker et al. 2014). The complete chloroplast genome sequence was annotated using GeSeq (Tillich et al. 2017) with *Adenophora triphylla* reference chloroplast genome (NCBI accession number, MG764158) and manual curation by Artemis annotation tool (Rutherford et al. 2000) with NCBI BLASTN searches.

*A. triphylla* chloroplast genome is 2,39,431 bp in length (GenBank accession no. MT649408), and has a circular structure with a large single-copy (LSC) region of 1,78,906 bp, a small single-copy (SSC) region of 55,819 bp, and a pair of 2,353 bp inverted repeats (IRa/IRb). A total of 108 genes were predicted in the genome, including 74 protein-coding genes, 30 transfer RNA genes, and 4 ribosomal RNA genes. Overall GC contents of chloroplast genome is 37.71%. Another *A. triphylla* chloroplast genome (MG764158) had already been published in the NCBI database. However, the size of the previously assembled and our assembled chloroplast genome was quite different, at 154,223 and 2,39,431 bp, respectively. This discrepancy in genome size was assumed to be due to the various repetitive sequences even though same species.

**Figure 1.**
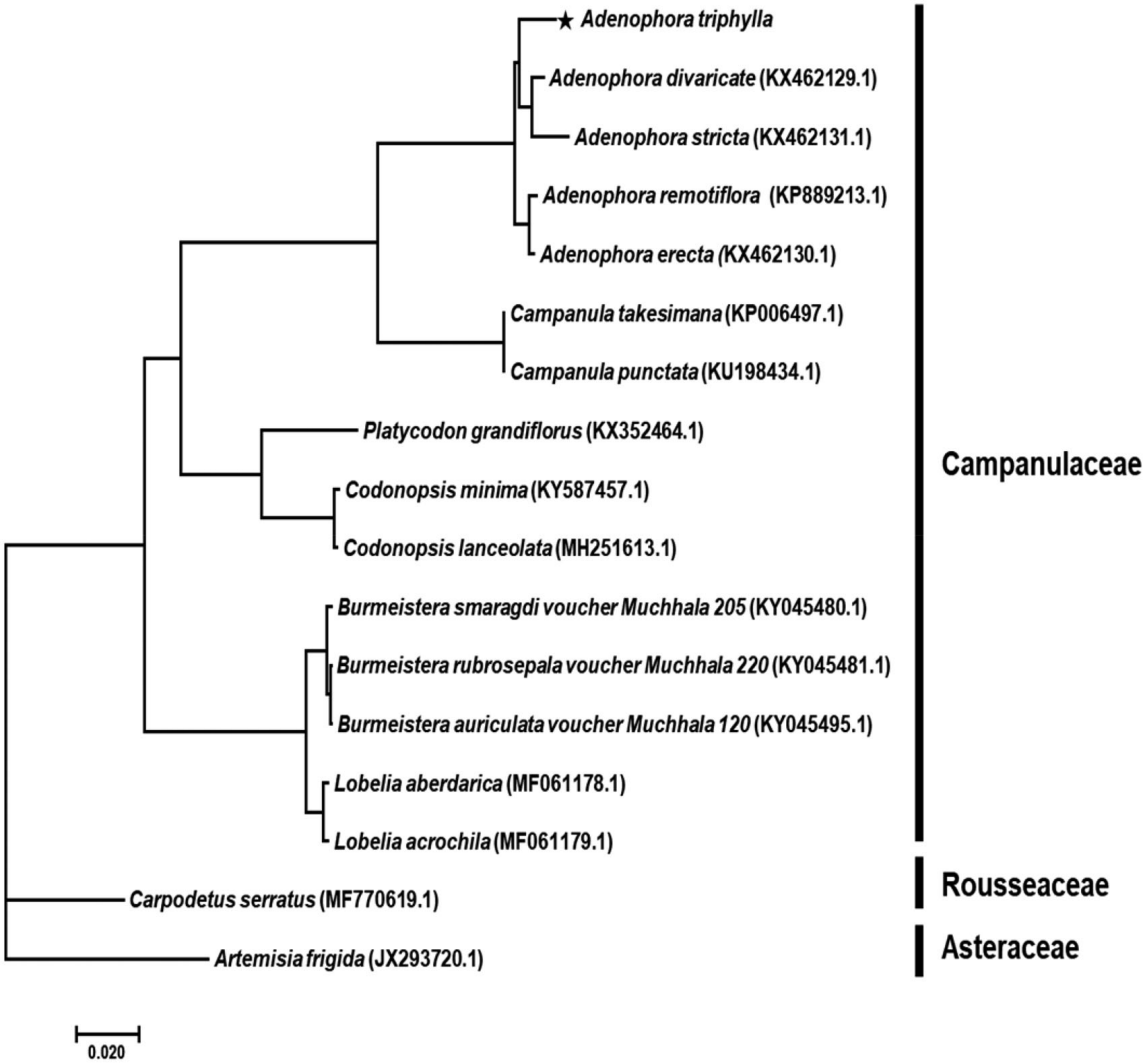
Phylogenetic tree of *Adenophora triphylla* with related taxa classification. The 68 protein-coding sequences in chloroplast genome were aligned. Bayesian inference (BI) tree constructed using the GTR + I + Γ model, and Markov Chain Monte Carlo (MCMC) chains were run for 10 million generations. All posterior probability value was 1.

A phylogenetic analysis was performed comparing the complete chloroplast genome against 14 *Campanulaceae* family species, including the 2 outgroups. Bayesian inference (BI) phylogenetic analysis was constructed based on multiple alignment of 68 protein-coding sequences by MrBayes 3.2.7 (Huelsenbeck and Ronquist 2001) using GTR + I + Γ nucleotide substitution model as for best model. The phylogenetic tree indicated that *A. triphylla* was belong to *Adenophora* genus as a sister group and most closely related to the *A. divaricate* (Figure 1). The outgroups were set *Carpodetus serratus* in *Rousseaceae* family and *Artemisia frigida* in *Asteraceae* family. This study will contribute to resolve problems related to the phylogeny of *Adenophora* genus.

## Geolocation

The genomic DNA sample used for sequencing was obtained from the National Institute of Horticultural and Herbal Science (geographic coordinate: N36°94′38″, E127°75′33″).

## Data Availability

The data that newly obtained at this study are available in the NCBI under accession number of MT649408 (https://www.ncbi.nlm.nih.gov/nuccore/MT649408/).
